# MHC class I-dressing is mediated via phosphatidylserine recognition and is enhanced by polyI:C

**DOI:** 10.1016/j.isci.2024.109704

**Published:** 2024-04-10

**Authors:** Arisa Hori, Saori Toyoura, Miyu Fujiwara, Ren Taniguchi, Yasutaka Kano, Tomoyoshi Yamano, Rikinari Hanayama, Masafumi Nakayama

**Affiliations:** 1 Laboratory of Immunology and Microbiology, College of Pharmaceutical Sciences, Ritsumeikan University, Kusatsu, Shiga 525-8577, Japan; 2 Department of Immunology, Kanazawa University Graduate School of Medical Sciences, 13-1 Takara-machi, Kanazawa, Ishikawa 920-8640, Japan; 3 WPI Nano Life Science Institute (NanoLSI), Kanazawa University, Kakuma-machi, Kanazawa, Ishikawa 920-1192, Japan; 4 Research Center for Animal Life Science, Shiga University of Medical Sciences, Seta, Tsukinowa-cho, Otsu, Shiga 520-2192, Japan

**Keywords:** Immunology, Components of the immune system, Cell biology

## Abstract

In addition to cross-presentation, cross-dressing plays an important role in the induction of CD8^+^ T cell immunity. In the process of cross-dressing, conventional dendritic cells (DCs) acquire major histocompatibility complex class I (MHCI) from other cells and subsequently prime CD8^+^ T cells via the pre-formed antigen-MHCI complexes without antigen processing. However, the mechanisms underlying the cross-dressing pathway, as well as the relative contributions of cross-presentation and cross-dressing to CD8^+^ T cell priming are not fully understood. Here, we demonstrate that DCs rapidly acquire MHCI-containing membrane fragments from dead cells via the phosphatidylserine recognition-dependent mechanism for cross-dressing. The MHCI dressing is enhanced by a TLR3 ligand polyinosinic-polycytidylic acid (polyI:C). Further, polyI:C promotes not only cross-presentation but also cross-dressing *in vivo*. Taken together, these results suggest that cross-dressing as well as cross-presentation is involved in inflammatory diseases associated with cell death and type I IFN production.

## Highlights

• Mouse splenic DCs acquire dead cell-derived MHCI via PtdSer recognition
• MHCI-containing vesicles are associated with the DC plasma membrane
• PolyI:C enhances MHCI dressing
• PolyI:C promotes not only cross-presentation but also cross-dressing *in vivo*

## Graphical abstract

**Figure:**
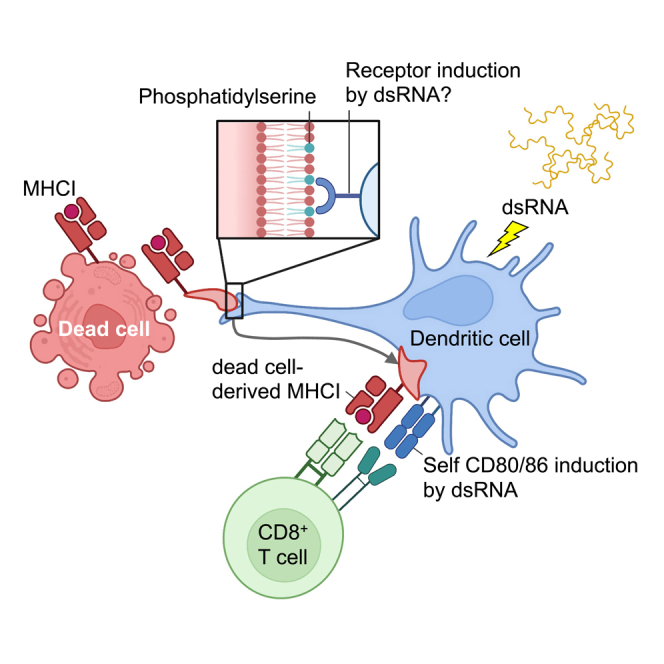

## Introduction

Exogenous antigen presentation by major histocompatibility complex class I (MHCI) on dendritic cells (DCs) is essential for CD8^+^ T cell-mediated immunity against tumor and viral antigens.^1^^,^^2^^,^^3^ To this end, a subset of conventional DCs (XCR1^+^ CD8α^+^ CD172a^−^ CD11b^−^ CD11c^+^ cells) called type 1 DCs (DC1s)^4^^,^^5^ can engulf dead cells (efferocytose), process cell-associated proteins, and present these exogenous antigens on MHCI, which is called cross-presentation.^1^^,^^2^^,^^3^ Although the other cDC subset (XCR1^-^ CD8α^−^ CD172a^+^ CD11b^+^ CD11c^+^ cells) called type 2 DCs (DC2s)^4^^,^^5^ present exogenous antigens on MHC class II for CD4^+^ T cell priming, DC2s can neither efferocytose nor present such exogenous antigens on MHCI.^1^^,^^2^^,^^3^ Under pathological conditions, DCs are likely stimulated by pathogen-derived Toll-like receptor (TLR) ligands and cytokines. Of note, TLR3 is expressed on DC1s but not DC2s.^4^^,^^5^ Thus, TLR3 ligands such as polyinosinic-polycytidylic acid (poly I:C), a synthetic dsRNA or viral-derived dsRNA induce DC1 maturation, upregulation of cross-presentation, and type I IFN (IFN-I) production,^6^^,^^7^ leading to control of viral infection.^3^ In contrast, pretreatment of TLR ligands reportedly inhibits the subsequent human DC efferocytosis^8^ and mouse cross-presentation.^9^ Taken together, this indicates that TLR signals transiently enhance cross-presentation and thereafter impair this process.^7^^,^^10^ Further, in a mouse tumor model, poly I:C therapy reportedly induces an anti-tumor effect independent of DC1 cross-presentation.^11^ Thus, this suggests the existence of another antigen presenting pathway for CD8^+^ T cell priming, especially under inflammatory conditions.

There is increasing evidence from mouse models that DCs are able to acquire MHCI from other cells and to use such pre-formed antigen-MHCI complexes to prime CD8^+^ T cells through a process referred to as cross-dressing.^2^^,^^12^^,^^13^^,^^14^ Of note, two recent studies using mouse models have shown that cross-dressing is essential for anti-tumor immunity.^15^^,^^16^ The initial step toward cross-dressing is DC acquisition of MHCI from other cells, referred to as “MHCI dressing” here, which is considered to be mediated either through cell-cell contact-dependent trogocytosis^12^^,^^17^ or via cell-cell contact-independent extracellular vesicles.^18^^,^^19^ However, it remains unknown how DCs recognize other cells and/or membrane vesicles to acquire MHCI, and how DCs express the acquired MHCI on their plasma membrane. It has been proposed that MHC dressing is a consequence of antigen uptake^20^^,^^21^ and that DCs internalize MHCI-containing cells and/or membrane fragments, some of which are recycled to the plasma membrane via the endogenous MHCI recycling pathway.^15^^,^^21^ However, this mechanism has not yet been confirmed. Furthermore, the relative contributions of cross-presentation and cross-dressing to CD8^+^ T cell priming are also not fully understood. It should be noted that the mechanism may differ between steady state and inflammatory conditions. In this study, we address the aforementioned questions using a simple model with MHCI H-2K^b^ (K^b^)^+^ and K^b−^ donor cells and recipient DCs and demonstrate the mechanism underlying the formation of the cross-dressing pathway.

## Results

### DCs rapidly acquire MHCI from dead cells upon PtdSer recognition

**Figure 1:**
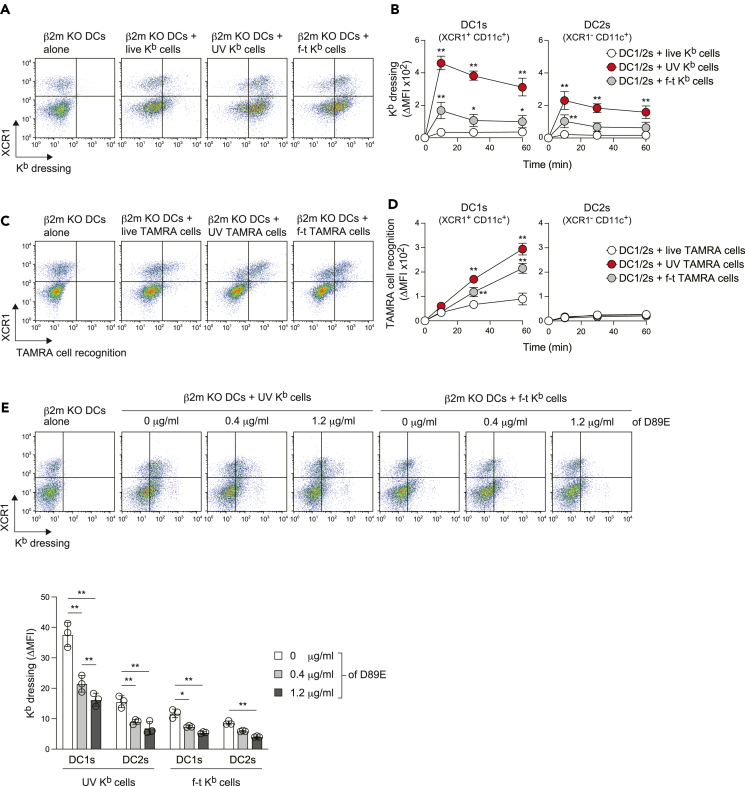
DCs rapidly acquire MHCI from dead cells upon PtdSer recognition (A and B) β2m KO mouse splenic DCs (1 × 10^5^) were cultured with live, UV-irradiated, or freeze-thawed (f-t) WT MHCI H-2K^b^ (K^b^) mouse splenocytes (5 × 10^5^ each) for 10 min (A) or the indicated periods (B) in a microtube. In (A), splenic DCs (CD11c^+^ cells) were gated as shown in Figure S1A and the K^b^ acquisition was analyzed by flow cytometry. In (B), the median fluorescence intensity (MFI) of K^b^ on DC1s (XCR1^+^ CD11c^+^) and DC2s (XCR1^-^ CD11c^+^) was analyzed, and the ΔMFI was calculated by subtracting MFI of K^b^ on each subset from MFI of K^b^ on the untreated subset as in Figure S1D. Data are shown as mean ± SD (*n* = 3 independent pools, where 2 mouse spleens were pooled; with a total of 6 mice per group) in (B). ∗*p* < 0.05, ∗∗*p* < 0.01 compared with live cells at each time point, by two-way ANOVA. (C and D) β2m KO mouse splenic DCs (1 × 10^5^) were cultured with tetramethylrhodamine (TAMRA)-labeled live, UV-irradiated, or f-t K^b^ splenocytes (5 × 10^5^ each) for 60 min (C) or the indicated time periods (D) in a microtube. The recognition (binding and engulfment) of TAMRA-labeled cells by DC1s and DC2s was analyzed by flow cytometry. The ΔMFI in (D) was calculated as in Figure S1D. Data are shown as mean ± SD (*n* = 3 independent pools, where 2 mouse LNs were pooled; with a total of 6 mice per group) in (D). ∗∗*p* < 0.01 compared with live cells at each time point, by two-way ANOVA. (E) UV-irradiated or f-t K^b^ mouse splenocytes pretreated with the indicated concentration of MFG-E8 mutant D89E for 30 min, were cultured with β2m KO mouse splenic DCs. K^b^ acquisition by DC1s and DC2s was analyzed as in (A). The graph shows individual value (dots), mean (columns), and SD (error bars) (*n* = 3 independent pools, where 2 mouse LNs were pooled; with a total of 6 mice per group). ∗*p* < 0.05, ∗∗*p* < 0.01, by two-way ANOVA. See also Figures S1 and S2.

We first addressed whether DCs are able to acquire MHCI from syngeneic live and dead cells. To this end, we used beta 2-microglobulin (β2m) knockout (KO) B6 mouse splenic DCs, which do not express any MHCI on their cell surface as recipient cells.^22^ By gradient density centrifugation followed by magnetic-activated cell sorting using anti-CD11c microbeads, we purified splenic DCs (>92% CD11c^+^) containing two subsets: DC1s (XCR1^+^ CD8α^+^ CD11b^−^ CD172a^−^ CD11c^+^) and DC2s (XCR1^-^ CD8α^−^ CD11b^+^ CD172a^+^ CD11c^+^)^4^^,^^5^ (Figure S1A). Thus, XCR1 or CD8α was used as a marker for DC1s. As donor cells, we used wild-type (WT) B6 mouse splenocytes that highly express K^b^ on their cell surface. We induced cell death by ultraviolet (UV) irradiation or freeze-thawing (f-t). Morphologically, UV irradiation resulted in the induction of Annexin V^+^ propidium iodide (PI)^-^ and Annexin V^+^ PI^+^ cells with chromatin condensation, indicative of late apoptotic cells. On one hand, f-t led all cells to become Annexin V^+^ PI^+^ with cell swelling, characteristic of accidental necrotic cells (Figures S1B and S1C). These cells were incubated with β2m KO DCs and the kinetic flow cytometric analysis showed that both DC1s and DC2s acquired K^b^ from UV-irradiated cells more efficiently than from f-t-treated cells, with both reaching maximum levels at around 10 min and the exogenous K^b^ protein being maintained on DCs until 60 min (Figures 1A, 1B, S1D, and S1E). Fluorescence microscopy showed that β2m KO DC plasma membranes are K^b^ positive (Figure S1F). Of note, DC1s acquired K^b^ more efficiently than did DC2s whereas neither DC1s nor DC2s efficiently acquired K^b^ from live cells (Figures 1A and 1B). When DCs and UV-irradiated cells were cultured in a transwell chamber, K^b^ acquisition was not observed (Figure S1E), indicating cells must be adjacent for it to occur. Taken together, these results indicate that both DC1s and DC2s rapidly acquire MHCI from neighboring dead cells.

We next cultured DCs with dead cells labeled with pH-insensitive tetramethylrhodamine (TAMRA) to assess the dead cell recognition (binding/engulfment)^23^ by DCs. Consistent with previous reports,^23^^,^^24^ the recognition of dead cells was observed in DC1s but not in DC2s (Figures 1C and 1D). Of note, these gradually increased until 60 min (Figures 1C and 1D). Similar kinetics were observed when dead cells were labeled with pH-sensitive CypHer5e^25^ fluorescent dye, an indicator of efferocytosis (Figure S1G). This was in contrast to the rapid MHCI dressing (Figures 1A and 1B). Taken together, these results suggest the MHCI dressing is not a consequence of efferocytosis. Given that DC1 efferocytosis is mediated via phosphatidylserine (PtdSer) recognition,^23^^,^^26^^,^^27^ MHCI dressing may occur in a PtdSer recognition-independent manner.

PtdSer exposure has recently been considered to occur not only in apoptotic cells but also in non-apoptotic cells such as necrotic cells.^28^^,^^29^ Thus, we next examined the effects of D89E, a point mutant of milk fat globule epidermal growth factor VIII (MFG-E8) that masks PtdSer on dead cells,^30^^,^^31^ on MHCI dressing. Unexpectedly, D89E significantly inhibited all MHCI dressing in a dose-dependent manner, irrespective of DC subsets and dead cell morphologies (Figure 1E). These results suggest that PtdSer recognition drives the bystander transfer of MHCI-containing membrane fragments to both DC1s and DC2s. Of note, this result also suggests that DC2s may also have the ability to recognize PtdSer, despite lacking the efferocytosis function.

Conventional DCs express PtdSer receptors Tim4^32^^,^^33^ and Tim3^23^^,^^34^ whereas plasmacytoid DCs (pDCs) express Tim1.^23^^,^^35^ Here we confirmed that expression of Tim3 is higher on DC1s than on DC2s and that Tim4 is faintly expressed both on DC1s and DC2s (Figure S2A). We next addressed whether Tim1 and Tim4 double-knockout (Tim1/4 DKO) B6 (K^b^) mouse DCs^35^ fail to acquire allogeneic K^d^ from BALB/c mouse apoptotic splenocytes. However, the level of K^d^ dressing was not significantly different between WT and Tim1/4 DKO DCs, indicating MHCI dressing is Tim1- and Tim4-independent (Figure S2B). Genetic deletion of Tim1 and Tim4 did not alter the Tim3 expression level (Figure S2A). As we and others have reported previously,^23^^,^^27^ anti-Tim3 mAb significantly blocked DC1 recognition of apoptotic cells (Figure S2C); however, it did not block MHCI dressing at all (Figure S2B), suggesting that although Tim3 mediates DC1 efferocytosis, neither Tim3 nor Tim4 mediate MHCI dressing on DC1s or DC2s. We also assessed the expression level of Axl and MerTK, which are involved in PtdSer recognition and efferocytosis^36^^,^^37^; however, we did not observe marked expression of these receptors on DCs (Figure S2A), suggesting that neither Axl nor MerTK contribute to MHCI dressing. Taken together, these results suggest that while MHCI dressing and efferocytosis rely on PtdSer recognition, these pathways differ.

### MHCI-containing membrane fragments are associated with the DC plasma membrane

**Figure 2:**
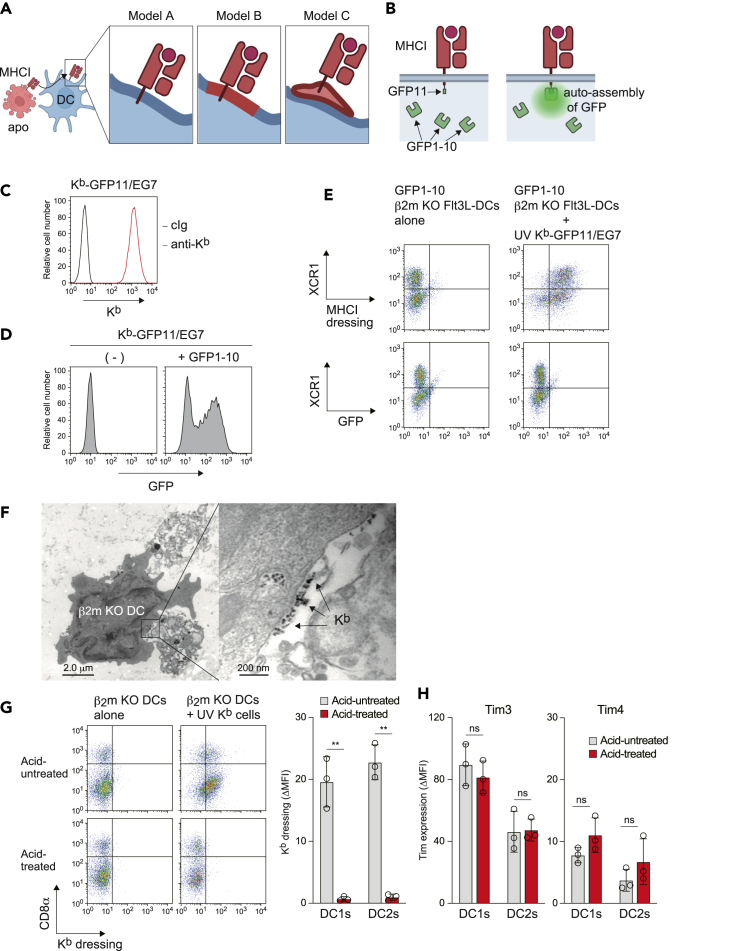
MHCI-containing membrane fragments are associated with the DC plasma membrane (A) The three hypothesized models of DC dressing of MHCI. In model A, donor MHCI is expressed on recipient DC plasma membrane. In model B, donor MHCI-containing membrane is fused with recipient DC plasma membrane. In model C, donor MHCI-containing membrane vesicle binds DC plasma membrane. (B) Principle of split-GFP system. The non-fluorescent GFP11 subunit is fused to the cytoplasmic domain of K^b^. In models A and B (A), donor cell-derived GFP11 is assembled with GFP1-10 in the cytosol of recipient DCs, resulting in the formation of fluorescent GFP chromophores. (C) K^b^ expression on K^b^-GFP11/EG7 cells was analyzed by flow cytometry. (D) GFP1-10 gene was transiently transduced into K^b^-GFP11/EG7 cells and the auto-assembly of GFP was analyzed by flow cytometry. (E) Flt3 ligand (Flt3L)-derived β2m KO mouse DCs were transduced with GFP1-10, and then these DCs (1 × 10^5^) were cultured with UV-irradiated K^b^-GFP11/EG7 cells (1 × 10^5^) for 10 min in a microtube. K^b^ acquisition and GFP expression in Flt3L-DCs (CD11c^+^ cells) were analyzed by flow cytometry. (F) β2m KO mouse splenic DCs cultured with UV-irradiated K^b^ cells were stained with biotinylated anti-K^b^, followed by streptavidin-10 nm gold particles. Cells were analyzed by transmission electron microscopy (TEM). See also Figure S2D. (G) β2m KO mouse splenic DCs were cultured with UV-irradiated K^b^ splenocytes as in Figure 1A. These cells were then cultured in PBS or citric acid buffer for 4 min at 20°C. K^b^ acquisition by splenic DCs (CD11c^+^ cells) was analyzed by flow cytometry. The ΔMFI of K^b^ on DC1s (CD8α^+^ CD11c^+^ cells) and DC2s (CD8α^−^ CD11c^+^ cells) was calculated as in Figure S1D. The graph shows individual value (dots), mean (columns), and SD (error bars) (*n* = 3 independent pools). ∗∗*p* < 0.01, two-way ANOVA. (H) β2m KO mouse splenic DCs were cultured in PBS or citric acid buffer for 4 min at 20°C. Expression of Tim3 and Tim4 on DC1s and DC2s were analyzed, and the ΔMFI was calculated as in Figure S1D. The graph shows individual value (dots), mean (columns), and SD (error bars) (*n* = 3 independent pools). ns, non-significant, two-way ANOVA.

There are three models of how exogenous MHCI proteins exit the DC surface (Figure 2A). In model A, MHCI is internalized once and re-expressed directly on the DC membrane, as proposed previously for MHCI dressing on T cells and DCs.^15^^,^^21^ In model B, MHCI-containing membrane fragments are fused with the DC membrane, as proposed for CD80 dressing on Treg cells.^38^ In model C, MHCI-containing membrane fragments are associated with the DC membrane, as proposed for MHCI dressing on NK cells.^39^^,^^40^ To investigate the possibility of models A and B, we performed a bi-molecular fluorescence complementation assay (Figure 2B).^41^ Specifically, the non-fluorescent GFP11 subunit was fused with the intracellular domain of K^b^ in mouse thymoma EG7 cells, resulting in K^b^-GFP11/EG7 cells (Figure 2C). Transient transduction of the non-fluorescent GFP1-10 subunit in K^b^-GFP11/EG7 cells produced a green fluorescence signal, confirming the spontaneous self-assembly of GFP1-10 and GFP11 subunits into GFP chromophores^41^ (Figure 2D). Due to the difficulty of genetic manipulation of splenic DCs, here we used Flt3 ligand-derived DCs (Flt3L-DCs), which contain DC1 and DC2 subsets.^42^ We engineered β2m KO Flt3L-DCs expressing the GFP1-10 subunit and confirmed the ability of these DCs to acquire MHCI from UV-irradiated EG7 cells (Figure 2E). If MHCI-dressing occurs as in model A or B, the EG7-derived GFP11 subunit would encounter the GFP1-10 subunit in the cytosol of Flt3L-DCs, resulting in production of the GFP signal. However, K^b^-dressed β2m KO Flt3L-DCs remained GFP negative (Figure 2E), disproving models A and B.

To investigate the possibility of model C, we next performed immunoelectron microscopy. β2m KO splenic DCs were cultured with UV-irradiated B6 K^b^ mouse splenocytes and repurified. Cells were then stained with biotinylated anti-K^b^, followed by 10 nm colloidal gold nanoparticle-labeled streptavidin. We found dead cell-engulfing DCs, on which K^b^-containing fragments were observed (Figure 2F). Likewise, several K^b^-containing vesicles were found to be associated with the DC plasma membrane (Figure S2D), supporting model C. Next, we conducted an acid-treatment assay.^39^^,^^43^ Mild citric acid treatment reportedly removes donor cell-derived proteins from the recipient cell surface without affecting recipient cell viability.^39^^,^^40^ Following acid treatment, the acquired MHCI on DCs disappeared (Figure 2G), further supporting model C, while endogenous Tim3 and Tim4 remained on DCs (Figure 2H). Taken together, these results suggest that K^b^-containing membrane vesicles are associated with the DC plasma membrane.

### Relative contributions of cross-dressing and cross-presentation to CD8^+^ T cell proliferation *in vitro*

**Figure 3:**
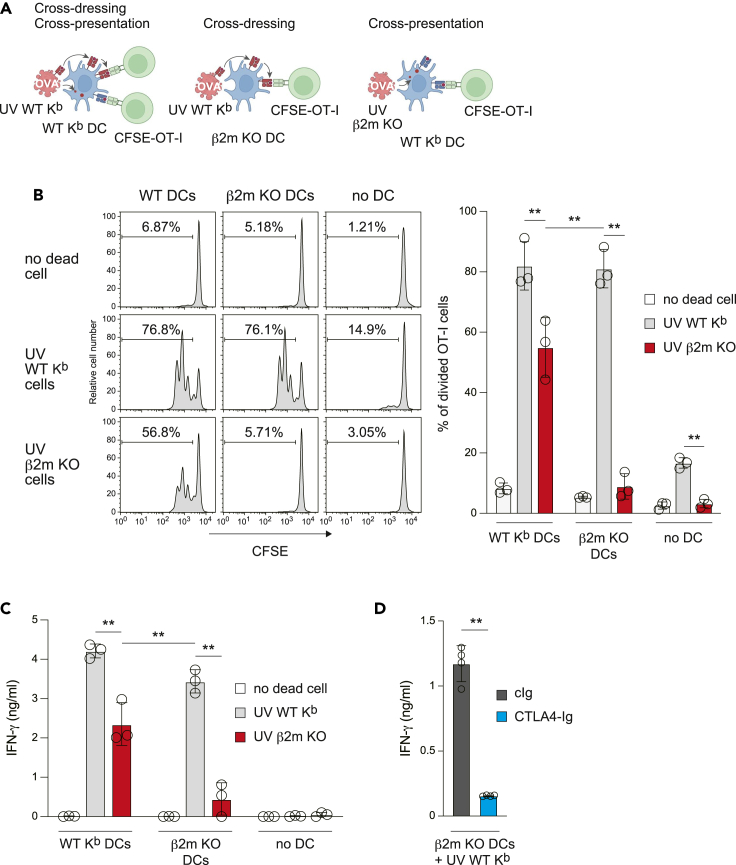
Relative contributions of cross-dressing and cross-presentation to CD8^+^ T cell proliferation *in vitro* (A) The experimental model of the *in vitro* antigen-presentation assay. (B) CFSE-labeled OT-I T cells (2 × 10^5^ per well) were co-cultured with indicated splenic DCs (1 × 10^5^ per well) with UV-irradiated and OVA-loaded splenocytes (1 × 10^5^ per well) in 96-well plates for 2 days. OT-I T cell proliferation (CFSE intensity in TCR Vβ5^+^ cells) was analyzed by flow cytometry. Numbers in histograms indicate the percentage of divided OT-I T cells. The graph shows individual value (dots), mean (columns), and SD (error bars) (*n* = 3 independent replicates). ∗∗*p* < 0.01, two-way ANOVA. (C) OT-I T cells were co-cultured with splenic DCs as in (B). Production of IFN-γ in the culture supernatants was measured by ELISA. Data are shown as individual value (dots), mean (columns), and SD (error bars) (*n* = 3 independent replicates). ∗∗*p* < 0.01, two-way ANOVA. (D) OT-I T cells (2 × 10^5^ per well) were co-cultured with β2m KO mouse DCs (1 × 10^5^ per well) and UV-irradiated and OVA-loaded WT mouse splenocytes (1 × 10^5^ per well) in the presence of CTLA4-Ig or control human IgG (cIg) (1 μg/mL each) in 96-well plates. Production of IFN-γ was analyzed as in (C). Data are shown as individual value (dots), mean (columns), and SD (error bars) (*n* = 4 independent replicates). ∗∗*p* < 0.01, unpaired two-tailed Student’s t test. See also Figure S3.

To address whether DCs use dead cell-derived MHCI for cross-dressing, we used splenic DCs, UV-irradiated splenocytes osmotically labeled with OVA protein, and OT-I T cells^44^ harboring a T cell receptor specific for OVA peptides presented by K^b^ (Figure 3A). When carboxyfluorescein diacetate succinimidyl ester (CFSE)-labeled OT-I T cells were co-cultured with K^b^ DCs without dead cells, the intensity of CFSE in OT-I T cells remained high, indicating no proliferation (Figure 3B). When OVA-associated K^b^ dead cells were added to K^b^ DCs, the intensity of CFSE in OT-I T cells decreased >70%, indicating vigorous OT-I T cell proliferation, which could be mediated both by cross-presentation and cross-dressing (Figure 3B). We next utilized a WT K^b^ dead cell and β2m KO DC culture system for a cross-dressing-specific assay, and a β2m KO dead cell and K^b^ DC culture system for a cross-presentation-specific assay. Each pathway effectively induced OT-I T cell proliferation, and IFN-γ production was induced by both cross-dressing as well as cross-presentation (Figure 3C). To exclude the possibility of the direct stimulation of OT-I T cells by K^b^ dead cells, we next addressed whether cytotoxic T lymphocyte antigen 4 (CTLA4) ectodomain fused with an IgG Fc domain (CTLA4-Ig)^45^ could block IFN-γ production. As expected, CTLA4-Ig significantly blocked the response (Figure 3D), indicating that OT-I T cell activation is not due to the direct presentation of K^b^ dead cells, but requires DC CD80/86 costimulation, confirming the T cell activation occurs via cross-dressing.

To further examine the *in vitro* cross-dressing ability of splenic DCs, we next used an OVA gene-transduced B16 (K^b^) melanoma cell line (OVA/B16) and β2m KO cells generated using the CRISPR/Cas9 system, in which we confirmed a 1 base insertion of the β2m open reading frame resulting in a frameshift mutation (Figure S3A). While endogenous K^b^ was not constitutively expressed on OVA/B16 cells, it was induced by IFN-γ (Figure S3B). β2m KO OVA/B16 cells respond to IFN-γ normally (Figure S3C); however, K^b^ induction was not observed (Figure S3B). These IFN-γ-stimulated and UV-irradiated OVA/B16 cells were added to WT or β2m KO DCs. As seen when cultured with dead K^b^ splenocytes osmotically labeled with OVA protein, cross-dressing as well as cross-presentation was observed (Figure S3D). IFN-γ production from OT-I T cells cocultured with β2m KO DCs with UV-irradiated OVA/B16 cells was significantly inhibited by CTLA4-Ig (Figure S3E), confirming the cellular response via cross-dressing. Further, D89E significantly blocked cross-dressing-mediated OT-I T cell proliferation (Figure S3F), which is consistent with PtdSer recognition-dependent MHCI acquisition (Figure 1E). Taken together, these results suggest that DCs recognize PtdSer on dead cells to acquire MHCI and use their own CD80/86 to prime CD8^+^ T cells.

### Relative contributions of cross-dressing and cross-presentation to CD8^+^ T cell proliferation *in vivo*

**Figure 4:**
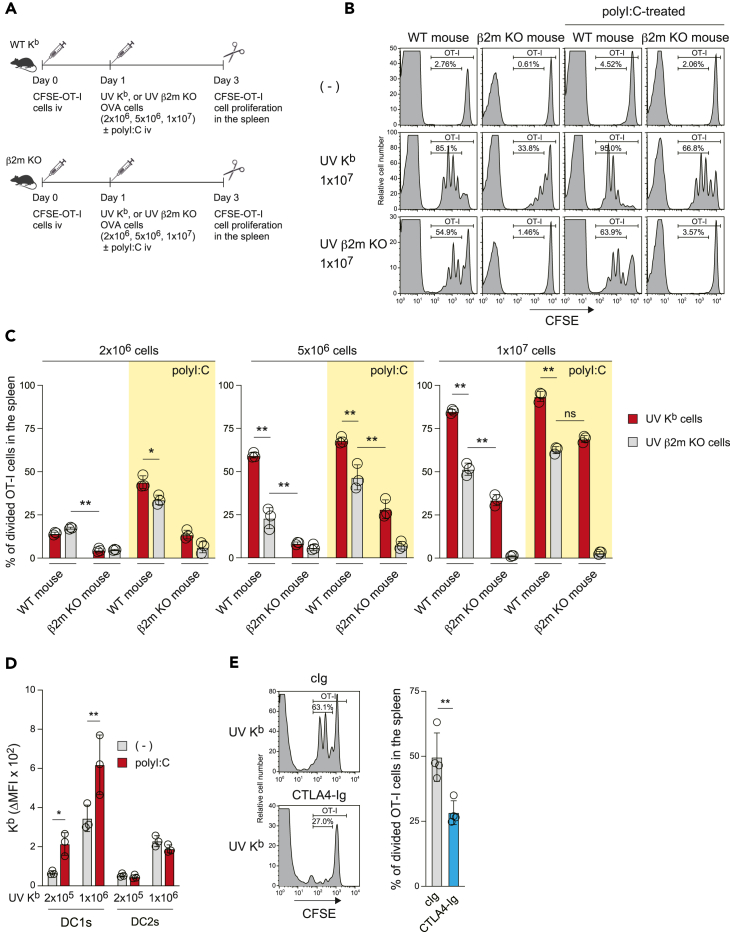
Relative contributions of cross-dressing and cross-presentation to CD8^+^ T cell proliferation *in vivo* (A) Experimental procedure for the *in vivo* antigen-presentation assay. (B and C) WT K^b^ or β2m KO mice were treated as in (A). OT-I T cell proliferation (CFSE intensity in TCR Vβ5^+^ cells) in the spleen was analyzed by flow cytometry. In (B), numbers in histograms indicate the percentage of divided OT-I T cells in mice injected i.v. with 1 × 10^7^ splenocytes. Histograms of the 2 × 10^6^ or 5 × 10^6^ groups, along with the gating strategy, are shown in Figure S4. In (C), the graph indicates individual values (dots), mean (columns), and SD (error bars) from *n* = 3 mice. Yellow boxes indicate polyI:C-treated mouse groups. ∗∗*p* < 0.01; ns, non-significant, two-way ANOVA. (D) β2m KO mice were injected intraperitoneally (i.p.) with PBS or polyI:C (200 μg/mouse). The following day, mouse splenic DCs were prepared and cultured (1 × 10^5^ each) with the indicated number of UV-irradiated K^b^ splenocytes for 10 min (D) in a microtube. The K^b^ dressing was analyzed by flow cytometry as in Figure S5. The graph indicates individual values (dots), mean (columns), and SD (error bars) from *n* = 3 independent pools. ∗*p* < 0.05, ∗∗*p* < 0.01, two-way ANOVA. (E) β2m KO mice adoptively transferred with CFSE-labeled OT-I T cells were injected i.p. with cIg or CTLA4-Ig (Abatacept: 300 μg/mouse each). The following day, mice were treated with UV-irradiated and OVA-loaded K^b^ splenocytes (1 × 10^7^) with polyI:C (200 μg/mouse). The polyI:C-induced cross-dressing was then analyzed as in (B). The graph indicates individual values (dots), mean (columns), and SD (error bars) from *n* = 4 mice. ∗∗*p* < 0.01, unpaired two-tailed Student’s t test.

The relative contributions of cross-presentation and cross-dressing *in vivo* to CD8^+^ T cell priming were investigated using WT K^b^ and β2m KO mice receiving CFSE-labeled OT-I T cells, followed by WT K^b^ and β2m KO dead cells (Figure 4A). Vigorous OT-I T cell proliferation was observed in WT mice injected with WT dead cells in a manner dependent on the number of dead cells, where both cross-presentation and cross-dressing occur (Figures 4B and 4C). Likewise, in WT mice injected with β2m KO dead cells, where cross-presentation but not cross-dressing occurs, we observed vigorous proliferation of OT-I T cells, although the amount of proliferation was lower than in WT mice injected with WT dead cells (Figures 4B, 4C, and S4). In β2m KO mice injected with WT dead cells, where cross-dressing but not cross-presentation occurs, the level of OT-I T cell proliferation was even lower than that observed in WT mice injected with β2m KO dead cells (Figures 4B, 4C, and S4). This contrasts with the strong *in vitro* effect of cross-dressing (Figures 3B and 3C). Taken together, these results suggest that CD8^+^ T cell priming is primarily mediated by cross-presentation rather than cross-dressing *in vivo* at steady state, which is consistent with early reports demonstrating the *in vivo* cross-presentation pathway.^46^^,^^47^

To activate DC1s *in vivo*, we next injected mice intravenously with polyI:C, a TLR3 ligand selectively expressed on DC1s.^6^^,^^48^^,^^49^ As reported previously,^6^ polyI:C enhanced the cross-presentation pathway as seen in WT mice injected with polyI:C and β2m KO dead cells, highlighted in yellow boxes in Figure 4C. Interestingly, polyI:C also strongly induced the cross-dressing pathway as seen by vigorous proliferation of OT-I T cells in β2m KO mice injected with polyI:C and WT dead cells, in a manner dependent on the number of dead cells (Figures 4C and S4). We also found that polyI:C significantly enhanced the MHCI dressing activity of DC1s in a manner dependent on the number of dead cells (Figures 4D and S5A). The injection of polyI:C induced DC maturation as indicated by enhanced expression of CD80/86 on the cell surface (Figure S5B). These results suggest that matured DC1s gain MHCI dressing activity. Further, *in vivo* treatment of CTLA4-Ig (Abatacept) significantly blocked the OT-I T cell proliferation in β2m KO mice injected with polyI:C and WT dead cells, confirming that the enhanced T cell proliferation occurs in response to cross-dressing (Figure 4E). Taken together, these results suggest that not only cross-presentation but also cross-dressing plays an important role *in vivo* under inflammatory conditions.

### PolyI:C enhances cross-dressing with tumor cell-derived MHCI

**Figure 5:**
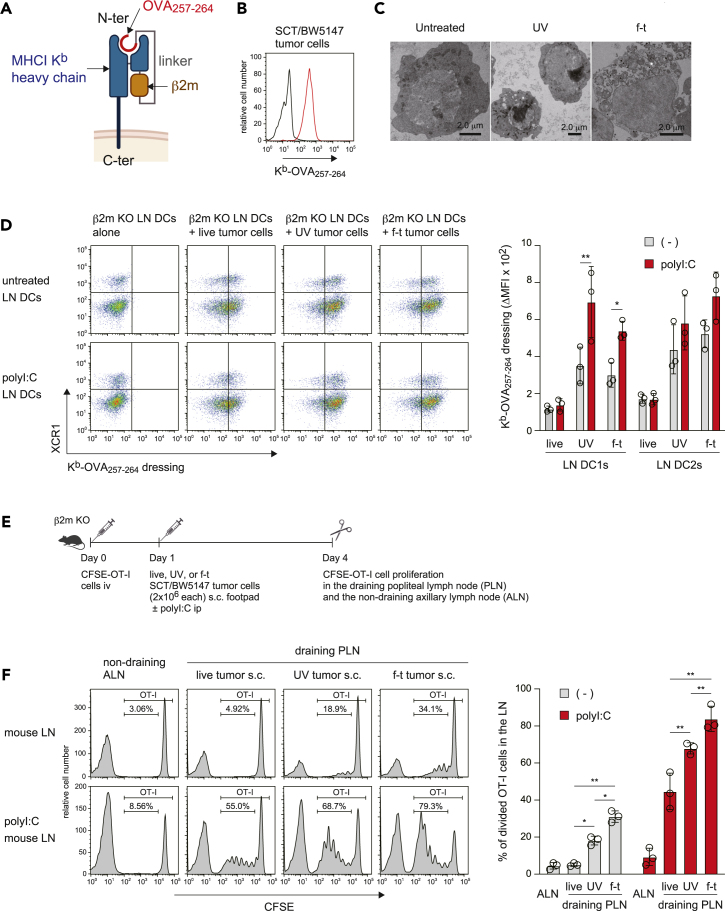
PolyI:C drives cross-dressing with tumor cell-derived MHCI (A) Schematic diagram of single-chain trimers (SCT) engineered with OVA_257-264_ peptide, β2m, and K^b^. (B) H-2K^k^ mouse T lymphoma cell line BW5147 cells were stably transduced with SCT, and the expression of SCT on the resulting SCT/BW5147 cells was analyzed by flow cytometry. Black and red histograms indicate cIg and 25-D1.16 mAb, respectively. (C) Morphology of live, UV-irradiated, or f-t SCT/BW5147 cells was analyzed by TEM. (D) β2m KO mice were injected i.p. with PBS or polyI:C (200 μg/mouse). The following day, mouse axillary, inguinal, and popliteal lymph nodes (LNs) were pooled together, and DCs were subsequently purified from the pooled LNs. These LN DCs (1 × 10^5^ each) were cultured with live, UV-irradiated, or f-t SCT/BW5147 tumor cells (2 × 10^5^ each) for 10 min in a microtube. The SCT acquisition by LN DCs (CD11c^+^ cells) was analyzed by flow cytometry as in Figure S5A. The graph indicates individual values (dots), mean (columns), and SD (error bars) from *n* = 3 independent pools. ∗*p* < 0.05, ∗∗*p* < 0.01, two-way ANOVA. (E) Experimental procedure for the *in vivo* cross-dressing assay. (F) β2m KO mice were treated as in (E). OT-I T cell proliferation (CFSE intensity in TCR Vβ5^+^ cells) in the LN was analyzed by flow cytometry. The graph indicates individual values (dots), mean (columns), and SD (error bars) from *n* = 3 mice. ∗*p* < 0.05, ∗∗*p* < 0.01, two-way ANOVA.

Finally, we addressed whether poly I:C enhances cross-dressing in draining lymph nodes (LNs) of mice subcutaneously injected with tumor cells. To focus on the donor cell-derived MHCI-peptide complexes as the sole antigen, we generated the H-2K^k^ mouse T lymphoma cell line BW5147 cells stably expressing single-chain trimers (SCT) engineered with OVA_257-264_ peptide, β2m, and K^b^ (SCT/BW5147 cells), with the antigen anchored to the plasma membrane (Figures 5A and 5B). To further address the effect of donor cell status (live, apoptotic, or necrotic) on cross-dressing, we treated these cells with UV irradiation or f-t, resulting in apoptotic or necrotic cells, respectively, as observed by TEM (Figure 5C). LN DCs effectively acquired K^b^-OVA_257-264_ from apoptotic as well as necrotic SCT/BW5147 tumor cells, and to a lesser degree, even from live tumor cells (Figure 5D). A marked difference of MHCI dressing between DC1s and DC2s, or between apoptotic and necrotic cells, was not observed (Figure 5D). Of note, polyI:C enhanced LN DC1 acquisition of K^b^-OVA_257-264_ from both apoptotic and necrotic tumor cells (Figure 5D), in a similar fashion to splenic DC acquisition of MHCI from dead splenocytes (Figures 4D and S5A). These results suggest that polyI:C enhances MHCI-dressing activity of both LN and splenic DC1s.

β2m KO mice receiving CFSE-labeled OT-I T cells were then injected subcutaneously with live, apoptotic, or necrotic SCT/BW5147 tumor cells into the footpad, where cross-dressing may occur in the draining popliteal LNs (PLNs) (Figure 5E). In these mice, a slight proliferation of OT-I T cells was observed in the draining PLNs of mice injected with apoptotic or necrotic but not live tumor cells (Figure 5F), suggesting that apoptotic and necrotic cells but not live cells induced cross-dressing. Necrotic cells had a stronger effect than apoptotic cells (Figure 5F). More importantly, polyI:C enhanced cross-dressing with apoptotic, necrotic, and even live tumor-derived MHCI (Figure 5F), although polyI:C did not enhance the live tumor-derived MHCI dressing on DC1s (Figure 5D). The enhanced cross-dressing with live tumor-derived MHCI may be ascribed to up-regulation of CD80/86 expression. Taken together, these results suggest that DCs acquire MHCI more efficiently from dead cells than live cells, and this is further enhanced by polyI:C, leading to strong cross-dressing.

## Discussion

A number of early reports of cross-presentation dismissed the possibility of cross-dressing.^2^^,^^3^^,^^7^ For example, studies by Kurts et al. utilizing an MHCI-mismatched bone marrow (BM) chimeric model clearly demonstrated that MHCI on BM-derived antigen-presenting cells is essential for exogenous antigen presentation *in vivo*.^46^^,^^47^ Consistent with these reports,^46^^,^^47^ here we observed that cross-presentation, rather than cross-dressing, preferentially contributes to CD8^+^ T cell priming *in vivo* under steady-state conditions. However, we also demonstrate that polyI:C enhances not only cross-presentation but also cross-dressing *in vivo*, which may explain why cross-dressing has frequently been observed in pathological conditions such as viral infection,^50^^,^^51^ cancer,^15^^,^^16^ and DNA vaccination,^52^ which are associated with the production of inflammatory cytokines including IFN-Is.

The relative contributions of DC1s and DC2s to cross-dressing remain controversial. For instance, studies on DNA vaccination^52^ or allograft transplantation^53^ have shown that cross-dressing is impaired in Batf3 KO mice specifically lacking DC1s.^4^ In contrast, in mouse models of viral infection, DC2s rather than DC1s effectively induce anti-viral CD8^+^ T cell proliferation.^50^^,^^51^ Two recent studies on syngeneic tumor-bearing mice have demonstrated that cross-dressing is essential for anti-tumor immunity but it is mediated via distinct DC subsets.^15^^,^^16^ Duong et al. identified the IFN-I-stimulated DC2s (ISG^+^ DCs) as cross-dressed DCs,^16^ whereas MacNabbs et al. demonstrated that DC1s are responsible for this process.^15^ The discrepancy between these two studies may be ascribed to use of different tumor models and/or DCs isolated from different site. Here, we observed that DC1s rather than DC2s acquired MHCI efficiently from dead splenocytes, while DC2s rather than DC1s appear to do this from dead tumor cells. Thus, whether DC1s or DC2s exert cross-dressing is context dependent. In mice injected with polyI:C, initial activation is likely limited to DC1s, as TLR3 is selectively expressed on DC1s.^6^^,^^54^ Indeed, we observed that polyI:C-treated mouse splenic DC1s, but not DC2s, show higher CD80 expression and enhanced MHCI dressing. These DC1s may subsequently activate DC2s by producing cytokines. This hypothesis is supported by previous findings that TLR3 signaling leads to IFN-I production^55^ and that ISG^+^ DCs contribute to cross-dressing.^16^ Therefore, under IFN-I-associated pathological conditions, both DC1s and DC2s may contribute to cross-dressing.

Regarding the mechanism underlying intercellular transfer of MHCI, several studies have shown that MHC dressing is mediated via extracellular vesicles such as exosomes.^13^^,^^56^ Exosome-mediated MHC dressing occurs even when donor cells and recipient cells are co-cultured at distance in a transwell plate.^57^^,^^58^ In contrast to these studies, we did not observe MHCI dressing in the transwell co-culture system, suggesting that this process may be mediated via cell-cell contact-dependent trogocytosis.^12^^,^^13^ However, it should be noted that capturing extracellular vesicles may require close proximity to DCs, even if direct cell-cell contact is not necessary. Thus, we cannot rule out the possibility that DCs acquire MHCI-containing membrane vesicles released from dead cells.

We here found that DCs acquire MHCI efficiently from both apoptotic and necrotic cells rather than live cells. This process, similar to efferocytosis, depends on PtdSer recognition. Thus, one might consider that MHCI dressing is associated with efferocytosis; however, this is likely not the case for several reasons: (1) MHCI dressing kinetically precedes efferocytosis, (2) While MHCI dressing occurs on both DC1s and DC2s, efferocytosis is executed solely by DC1s, (3) Tim3 blocking reduces efferocytosis but not MHCI dressing. Based on these observations, MHCI dressing may be executed by the polyI:C-inducible receptor(s) with low affinity to PtdSer, which is/are not sufficient to engulf whole dead cells. The receptor is probably constitutively expressed on DC2s that cannot engulf dead cells. In this context, PtdSer receptors Tim3 and Tim4^23^^,^^27^ and DNGR-1, a C-type lectin that recognizes dying cell-derived F-actin,^59^^,^^60^ are reportedly expressed on DC1s and to a lesser extent on DC2s, although we failed to observe the contribution of Tim3 and Tim4 to MHCI dressing. Axl^27^ and scavenger receptor SCARF1^61^ reportedly contribute to the indirect recognition of PtdSer on apoptotic cells by DCs. Of note, Duong et al. observed that ISG^+^ DCs express Axl.^16^ Here, we did not observe the expression of Axl or MerTK on unstimulated DCs; however, these receptors may be induced by polyI:C or IFN-Is. We are currently investigating the identity of PtdSer receptor for MHCI dressing.

The expression mode of donor cell-derived MHCI on recipient cell surface may also be context dependent. MacNabb et al. observed that exogenous peptide-MHCI complexes are internalized by DCs. Thus, they hypothesize that these internalized MHCI complexes are recycled to the plasma membrane via the endogenous MHCI recycling pathway.^15^ In contrast, we observed that MHCI-containing membrane fragments are merely associated with the DC plasma membrane, which is stripped by acid treatment. Of note, this association is stable enough to prime CD8^+^ T cells. In this study, the DCs acquire MHCI from dead cells within 60 min. Therefore, we cannot rule out the possibility that some of the MHCI may be recycled thereafter, as proposed by MacNabb et al.^15^ In this context, in acidic conditions of tumor microenvironments,^62^ DCs may no longer associate with MHC-containing vesicles on their surface. Instead, the alternative mechanism may exist for MHCI dressing.

In conclusion, our study shows that both DC1s and DC2s rapidly acquire MHCI from dead cells via PtdSer recognition. The MHCI-containing membrane vesicles are stably associated with the DC plasma membrane for cross-dressing. Importantly, polyI:C enhances cross-dressing, suggesting that under inflammatory conditions, cross-dressing may play an important role in CD8^+^ T cell priming.

### Limitations of the study

Although we found that recognition of PtdSer by DC1s and DC2s is essential for MHCI-dressing, neither Tim1, Tim3, nor Tim4 are involved in this process. Thus, the PtdSer receptor remains to be identified. Given that polyI:C enhances MHCI dressing, the polyI:C-TLR3 axis may lead to the induction the expression of low affinity PtdSer receptor, which is sufficient for MHCI dressing. During this process, MHCI may be internalized and re-expressed on recipient DCs. Identification of the PtdSer receptor will provide an understanding of how MHCI-dressing is facilitated by polyI:C. Further, gene targeting of the PtdSer receptor on DC1s and DC2s may produce a selective loss of the cross-dressing pathway, which would provide a better understanding of the physiological relevance of this pathway. We hypothesize that DC2s may have cross-dressing activity but require DC1 cell help under inflammatory conditions. Further studies using appropriate genetic mouse models specifically lacking each DC subset^4^ will be required to understand the relative contribution of DC1s and DC2s to cross-dressing.

## STAR★Methods

### Key resources table

**Table:** 

REAGENT or RESOURCE	SOURCE	IDENTIFIER
**Antibodies**		

FITC-hamster anti-mouse CD11c (clone N418)	BioLegend	Cat# 117306; RRID: AB_313774
FITC-mouse anti-mouse/rat XCR1 (clone ZET)	BioLegend	Cat# 148210; RRID: AB_2564366
PE-mouse anti-mouse H-2Kb (clone AF6-88.5)	BioLegend	Cat# 116507, RRID: AB_313734
PE-mouse anti-mouse H-2Kd (clone SF1-1.1)	BioLegend	Cat# 116607, RRID: AB_313742
PE-mouse anti-mouse H-2Kd/H-2Dd (clone 34-1-2S)	BioLegend	Cat# 114708, RRID: AB_313607
PE-mouse anti-mouse H-2Kb bound the peptide SIINFEKL (clone 25-D1.16S)	BioLegend	Cat# 141603, RRID: AB_10897938
PE-rat anti-mouse CD8α (clone 53–6.7)	BioLegend	Cat# 100707, RRID: AB_312716
PE-mouse anti-mouse/rat XCR1 (clone ZET)	BioLegend	Cat# 148203; RRID: AB_2563842
PE-rat anti-mouse/human CD11b (clone N1/70)	BioLegend	Cat# 101207, RRID: AB_312770
PE rat anti-mouse CD365/Tim1 (clone RMT1-4)	BioLegend	Cat# 119505, RRID: AB_2248167
PE rat anti-mouse CD366/Tim3 (clone RMT3-23)	BioLegend	Cat# 119703, RRID: AB_345377
PE rat anti-mouse Tim4 (clone RMT4-54)	BioLegend	Cat# 130005, RRID: AB_1227807
PE mouse anti-mouse TCR Vβ5.1/5.2 (clone MR9-4)	BioLegend	Cat# 139504, RRID: AB_10613279
PE-hamster anti-mouse CD80 (clone 16-10A1)	BioLegend	Cat# 104707, RRID: AB_313128
PE-rat anti-mouse CD86 (clone 1A17199A)	BioLegend	Cat# 159203, RRID: AB_2832567
PE-rat IgG2a, isotype control (clone RTK2758)	BioLegend	Cat# 400508; RRID: AB_326530
PE-rat IgG2b, isotype control (clone RTK4530)	BioLegend	Cat# 400608; RRID: AB_326551
PE-Hamster IgG (clone eBio299Arm)	eBioscience	Cat# 12-4888-81, RRID: AB_470073
PE/Cyanine7-rat anti-CD172a (clone P84)	BioLegend	Cat# 144007, RRID: AB_2563545
PerCP/Cyanine5.5-mouse anti-mouse/rat XCR1 (clone ZET)	BioLegend	Cat# 148207, RRID: AB_2564363
APC-rat anti-mouse CD8α (clone 53–6.7)	BioLegend	Cat# 100711, RRID: AB_312750
APC-hamster anti-mouse CD11c (clone N418)	BioLegend	Cat# 117309, RRID: AB_313778
APC-rat anti-mouse Axl (clone MAXL8DS)	Themo Fischer Scientific	Cat# 17-1084-82, RRID: AB_2734848
APC-rat anti-mouse MerTK (clone 2B10C42)	BioLegend	Cat# 151507, RRID: AB_2650738
Biotin-mouse anti-mouse H-2Kb (clone AF6-88.5)	BioLegend	Cat# 116503, RRID: AB_313730
Ultra-LEAF purified rat anti-mouse CD366/Tim3 (clone RMT3-23)	BioLegend	Cat# 119702, RRID: AB_2810361
Purified rat anti-mouse CD16/32 (clone 93)	BioLegend	Cat# 101302, RRID: AB_312800
Purified rat IgG2a (clone RTK2758)	BioLegend	Cat# 400503, RRID: AB_2783537
Purified human IgG	Thermo Fisher Scientific	Cat# 31154, RRID: AB_243591
Purified rabbit anti-phospho STAT1 (clone D4A7)	Cell Signaling Technology	Cat# 7649, RRID: AB_2798913
Purified rabbit anti-STAT1	Cell Signaling Technology	Cat# 9172, RRID: AB_2198300
Purified rabbit anti-β-actin	Cell Signaling Technology	Cat# 4967, RRID: AB_330288

**Bacterial and virus strains**		

*E. coli* TOP10 competent cells	Thermo Fisher Scientific	Cat# C404010
*E. coli* NEB Stable competent cells	New England BioLabs	Cat# C3040H

**Chemicals, peptides, and recombinant proteins**		

CFSE	Thermo Fisher Scientific	Cat# C34554
CypHer5E NHS ester	Cytiva	Cat# PA15401
TAMRA	Thermo Fisher Scientific	Cat# C1171
CD11c microbeads	Miltenyi Biotech	Cat# 130-125-835
Anti-FITC microbeads	Miltenyi Biotech	Cat# 130-048-701
Optiprep	Sigma-Aldrich	Cat# D1556
Recombinant human Flt3L	BioLegend	Cat# 550604
Recombinant mouse IFN-γ	BioLegend	Cat# 575306
Strepavidin-10 nm gold particles	Merck	Cat# S9059
DyLight594-streptavidin	BioLegend	Cat# 405222
Recombinant mouse CTLA4-Ig	BioLegend	Cat# 591806
Abatacept	Bristol Myers suibb	N/A
polyI:C	InvivoGen	Cat# Tlrl-picw

**Critical commercial assays**		

Mouse IFN-gamma DuoSet ELISA	R&D systems	Cat# DY485

**Experimental models: Cell lines**		

EG7 cells	ATCC	CRL-2113
B16 cells	ATCC	CRL-6322
BW5147 cells	ATCC	TIB-47
Lenti-X 293T cells	Takara Bio	632180

**Experimental models: Organisms/strains**		

Mouse: Wild type (WT): C57BL/6J	CLEA Japan	N/A
Mouse: Wild type (WT): BALB/c	CLEA Japan	N/A
Mouse: *β2m* KO: B6.129P-*B2m*^*tm1Unc*^/Dcr	The Jackson Laboratory	JAX# 002087
Mouse: OT-I: C57BL/6J-Tg (TcraTcrb)1100Mjb/J	The Jackson Laboratory	JAX# 003831
Mouse: Tim1 Tim4 DKO: C57BL/6J	Omori et al.,^35^ 2021	N/A

**Oligonucleotides**		

Mouse β2m gRNA	This paper	5′-ctggtgcttgtctcactgac-3′

**Recombinant DNA**		

pCAGGS-D89E-flag	Hanayama et al.,^30^ 2002	N/A
pQCXIP-GFP1-10	addgene	# 68715
pEF6/V5-His-TOPO	Thermo Fisher Scientific	#K961020
pHAGE-EF1aL-eGFP-W	Addgene	#126686
pEF6/V5-GFP1-10	This paper	N/A
pHAGE-EF1aL-GFP1-10	This paper	N/A
pQCXIP-BSR-GFP11	Addgene	#68716
pHAGE-EF1aL-H-2K1-GFP11	This paper	N/A
pMD2.G	Addgene	#12259
psPAX2	Addgene	#12260
pMXs-IRES-neo	Kitamura et al.,^63^ 2003	N/A
pMXs-IRES-neo-OVA	This paper	N/A
lentiCRISPR v2	Addgene	#52961
pMDLg/pRRE	Addgene	#12251
pRSV-Rev	Addgene	#12253
pHAGE-EF1aL-SCT	This paper	N/A

**Software and algorithms**		

BZ-X800 analyzer	Keyence	N/A
Prism 8	GraphPad	N/A
FlowJo version 10.7.1	BD Biosciences	N/A

### Resource availability

#### Lead contact

Further information and request for resources and reagents should be directed to and fulfilled by the Lead Contact (mnakayam@fc.ritsumei.ac.jp).

#### Materials availability

All materials generated in this study are available from the lead contact with a completed Materials Transfer Agreement.

#### Data and code availability

• All data reported in this paper will be shared by the lead contact upon request.
• This paper does not report original code.
• Any additional information required to reanalyze the data reported in this paper is available from the lead contact upon request.

### Experimental model and study participant details

#### Mice

WT C57BL/6 mice and BALB/c mice were obtained from CLEA Japan. β2m KO C57BL/6 mice and OT-I mice expressing OVA257-264/K^b^-specific TCR were obtained from The Jackson Laboratory. Female mice aged 8–12 weeks old were used for all experiments. Tim1/4 DKO C57BL/6 mice were generated previously.^35^ All mice were maintained under specific pathogen-free conditions according to the Guidelines of Proper Conduct of Animal Experiments (Science Council of Japan), and all protocols were approved by the institutional review committees of Ritsumeikan University (approved number BKC2020-041-1) and of Shiga University of Medical Sciences (approved numbers 2019-4-9 and 2022-6-12).

#### Cell culture

EG7 cells, B16 cells, and BW5147 cells (American Type Culture Collection [ATCC]) were maintained in complete RPMI-1640 (RPMI-1640 supplemented with 10% FBS, 100 U/mL penicillin, 100 mg/mL streptomycin, 2 mM glutamine, and 1 mM sodium pyruvate). Lenti-X 293T cells (Takara Bio) were maintained in complete DMEM.

### Method details

#### DCs

Mouse splenic and LN DCs were prepared according to our previous paper.^64^ In brief, mouse spleens were digested with 400 U/mL collagenase (034–22363, Wako Biochemicals) in the presence of 5 mM EDTA and separated into low-and high-density fractions on Optiprep density gradient medium (07820, STEMCELL technologies). Low-density cells were purified using anti-CD11c microbeads (130-125-835, Miltenyi Biotech). Flt3L-DCs were derived from mouse bone marrow cells cultured with recombinant human Flt3L (550604, BioLegend) according to a previous paper.^65^

#### Generation of cell lines

The OVA coding region (GenBank: V00383.1) was amplified from the OVA gene-transduced mouse T lymphoma cell line EG7 cells (ATCC). The cDNA was subcloned into pMXs-IRES-neo (pMXs-IN).^63^ The gene was retrovirally transduced into mouse melanoma cell line B16 cells (ATCC). To generate β2m KO OVA/B16 cells, mouse *β2m* gRNA 5′-ctggtgcttgtctcactgac-3′, designed using the software program CRISPRdirect,^66^ was subcloned into lentiCRISPRv2 vector, a gift from Feng Zhang (addgene #52961). The lentivirus was prepared and used to infect OVA/B16 cells according to our previous report.^67^ The mutant sequence is shown in Figure S3A. SCT encoding the leader sequence of β2m followed by SIINEKL sequence, a first linker of GGGAS (G_4_S)2, β2m sequence, the second liker of (G_4_S)4, and K^b^ sequence,^68^ was subcloned into pHAGE-EF1α lentiviral vector after removing EGFP from pHAGE-EF1aL-eGFP-W (Addgene plasmid #126686), resulting in the generation of SCT/pHAGE-EF1α vectors. The lentivirus was prepared and used to infect BW5147 cells according to our previous report.^67^ Cell lines used for the split-GFP system are described below.

#### Cell surface expression

DCs were preincubated with anti-CD16/32 mAb (clone 93, BioLegend), and were stained with the following mAbs: fluorescent isothiocyanate (FITC)-*anti*-CD11c (clone N418, BioLegend), FITC-anti-XCR1 (clone ZET, BioLegend), phycoerythrin (PE)-anti-CD11b (clone M1/70, BioLegend), PE-*anti*-CD8α (clone 53–6.7, BioLegend), PE-*anti*-Tim1 (clone RMT1-4, BioLegend), PE-*anti*-Tim3 (clone RMT3-23, BioLegend), PE-*anti*-Tim4 (clone RMT4-54, BioLegend), PE-*anti*-CD80 (clone 16-10A1, BioLegend), PE-*anti*-CD86 (clone GL-1, BioLegend), PE-Hamster IgG (clone eBio299Arm, eBioscience), PE-rat IgG2a (clone RTK2758, BioLegnd), PE-rat IgG2b (clone RTK4530, BioLegnd), PE/Cyanine7-*anti*-CD172a (clone P84, BioLegend), allophycocyanin (APC)-*anti*-CD8α (clone 53–6.7, BioLegend), APC-*anti*-Axl (clone MAXL8DS, Thermo Fischer Scientific), APC-*anti*-MerTK (clone 2B10C42, BioLegend).

#### Western blots

OVA/B16 cells and β2m KO OVA/B16 cells (2 × 10^6^ per well) were cultured in 6-well plates and stimulated with 50 ng/mL of IFN-γ (575306, BioLegend) for the indicated periods at 37°C. Cells were solubilized in RIPA buffer (1% Nonidet P-40, 50 mM Tris-HCl [pH 8.0], 150 mM NaCl, 0.5% deoxycholate, and 10% SDS) supplemented with a phosphatase inhibitor cocktail (Nacalai Tesque, Kyoto, Japan) and a protease inhibitor cocktail (Sigma-aldrich, St. Louis, MO). Cell lysates were separated by SDS-PAGE and transferred to a polyvinylidene difluoride membrane (Millipore, Billerica, MA) followed by detection with Abs against phospho-STAT1 (Tyr701) (clone D4A7, 7649, Cell Signaling Technology), total STAT1 (9172, Cell Signaling Technology), and β-actin (4967, Cell Signaling Technology). Membranes were developed with ECL prime western blotting detection reagent (GE Healthcare, Little Chalfont, United Kingdom) and analyzed with an OptimaShot CL-420D image analyzer (Wako, Osaka, Japan).

#### MHCI dressing

For fluorescent microscopy, β2m KO mouse splenic DCs (5 × 10^5^) were cultured with WT K^b^ mouse live splenocytes or UV (500 mJ/cm^2^)-irradiated splenocytes (2.5 × 10^6^ each) in a microtube for 10 min at 37°C. Then DCs were re-purified by using anti-CD11c MACS microbeads (130-125-835, Miltenyi Biotech) and stained with biotinylated anti-K^b^ mAb (clone AF6-88.5, BioLegend), followed by FITC-*anti*-CD11c mAb (clone N418, BioLegend) and DyLight594-streptavidin (405222, BioLegend). Cells were then analyzed using the optical sectioning microscope BZ-X800 (KEYENCE) equipped with a 100× objective lens.

For flow cytometry, β2m KO mouse DCs (1 × 10^5^) were cultured with the indicated number of live, UV (500 mJ/cm^2^)-irradiated, or f-t K^b^ mouse splenocytes or SCT/BW5147 cells in a microtube, 96-well flat-bottom cell culture plate, or 96-well transwell plate with a 0.4-μm pore membrane (3381, Corning) for the indicated periods at 37°C. Then cells were stained with the following mAbs: PE-*anti*-MHCI (clone 34-1-2s, BioLegend) or PE-*anti*-OVA_257-264_ peptide bound to K^b^ (clone 25-D1.16, BioLegend) with FITC-*anti*-XCR1 (clone ZET, BioLegend) and APC-anti-CD11c (clone N418, BioLegend), or with FITC-*anti*-CD11c (clone N418, BioLegend) and APC-*anti*-CD8α (clone 53–6.7, BioLegend). Cells were analyzed on an Accuri C6 Plus cytometer (BD Biosciences). Regarding MHCI trogocytosis between allogeneic cells, C57BL/6 (K^b^) WT or Tim1/4 DKO mouse splenic DCs (1 × 10^5^) were cultured with UV (500 mJ/cm^2^)-irradiated apoptotic BALB/c mouse (K^d^) splenocytes (1 × 10^5^) in a microtube for 10 min at 37°C. Then these cells were stained with FITC-*anti*-XCR1 mAb (clone ZET, BioLegend), APC-anti-CD11c mAb (clone N418, BioLegend), and PE-*anti*-K^d^ mAb (clone SF1-1.1, BioLegend). For masking PtdSer on dead cells, soluble D89E protein was produced by 293T cells transiently transfected with pCAGGS-D89E-flag.^30^^,^^31^ UV-irradiated or f-t cells were preincubated with the indicated concentration of D89E protein for 30 min at 37°C, and then cultured with DCs. Cells were analyzed on an Accuri C6 Plus cytometer (BD Biosciences).

#### Dead cell recognition and efferocytosis

Live, UV (500 mJ/cm^2^)-irradiated, and f-t cells were labeled with pH-insensitive TAMRA (C1171, Thermo Fischer Scientific) or pH-sensitive CypHer5E (PA15401, Cytiva) according to previous reports.^23^^,^^25^ β2m KO mouse splenic DCs (1 × 10^5^) were cultured with the indicated number of fluorescently labeled cells in a microtube for the indicated periods at 37°C. Then cells were stained with FITC-anti-XCR1 mAb (clone ZET, BioLegend) and APC-*anti*-CD11c mAb (clone N418, BioLegend), or with FITC-*anti*-CD11c (clone N418, BioLegend) and PE-*anti*-XCR1 mAb (clone ZET, BioLegend) or PE-anti-CD8α (clone 53–6.7, BioLegend). Cells were analyzed on an Accuri C6 Plus cytometer (BD Biosciences).

#### Split-GFP system

The coding region of non-fluorescent GFP1-10 subunit in pQCXIP-GFP1-10 (Addgene plasmid #68715) was subcloned into pEF6/V5-His-TOPO vector (Thermo Fisher Scientific) or pHAGE-EF1α lentiviral vector after removing EGFP from pHAGE-EF1aL-eGFP-W (Addgene plasmid #126686), resulting in the generation of GFP1-10/pEF6 and GFP1-10/pHAGE-EF1α vectors. The coding region of non-fluorescent GFP11 subunit in pQCXIP-BSR-GFP11 (Addgene plasmid #68716) was fused to the C-terminal cytoplasmic domain of H-2K1 (GeneBank: NM_001001892.3) that encodes MHCI K^b^ protein. The resulting H-2K1-GFP11 construct was then subcloned into the pHAGE-EF1α lentiviral vector. Subsequently, K^b^-GFP11 was transduced into EG7 cells. The K^b^-GFP11/EG7 cells were transiently transfected with GFP1-10/pEF6, and spontaneous self-assembly of GFP1-10 and GFP11 into fluorescent GFP chromophores in these cells was confirmed by flow cytometry. Then trogocytosis assay using the split-GFP system was performed according to a previous report.^41^ In brief, GFP1-10 was lentivirally transduced into β2m KO mouse Flt3L-DCs. The resulting GFP1-10/β2m KO Flt3L-DCs (1 × 10^5^) were cultured with UV (500 mJ/cm^2^)-irradiated K^b^-GFP11/EG7 cells (1 × 10^5^) in a microtube for 10 min at 37°C. Then cells were stained with PE-*anti*-MHCI mAb (clone 34-1-2s, BioLegend), PerCP/Cyanine5.5-*anti*-XCR1 (clone ZET, BioLegend) and APC-*anti*-CD11c mAb (clone N418, BioLegend). The spontaneous self-assembly of GFP1-10 and GFP11 into fluorescent GFP chromophores in Flt3L-DCs was analyzed on an Accuri C6 Plus cytometer (BD Biosciences).

#### Electron microscopy

For immunoelectron microscopy, β2m KO splenic DCs (3 × 10^6^) were cultured with UV (500 mJ/cm^2^)-irradiated apoptotic K^b^ mouse splenocytes (3 × 10^7^) in a microtube for 1 h at 37°C. Then DCs were re-purified using anti-CD11c microbeads (130-125-835, Miltenyi Biotech), and stained with biotinylated anti-K^b^ mAb (clone AF6-88.5, BioLegend), followed by streptavidin-10 nm gold particles (S9059, Merk). Cells were then embedded in iPGell (PG20-1, GenoStaff) according to the manufacturer’s instructions. The cell blocks were fixed with 4% formaldehyde and 2% glutaraldehyde in phosphate-buffered saline (PBS; pH7.4) overnight at 4°C, then were postfixed with 1% OsO_4_ in PBS for 2 h. Following dehydration in a series of graded concentrations of ethanol, the fixed cell blocks were embedded in epoxy resin (Luveak 812; Nacalai Tesque). Ultrathin sections (70-nm thickness) were prepared on an ultramicrotome (EM UC7; Leica). The sections were then stained with uranyl acetate and lead citrate and finally examined with an electron microscope (JEM-1400Flash, JEOL Ltd.). For the standard electron microscopy, live, UV (500 mJ/cm^2^)-irradiated, f-t splenocytes and SCT/BW5147 cells were fixed and analyzed as described above. TEM was performed at the Division of Electron Microscopic Study, Center for Anatomical Studies, Graduate School of Medicine, Kyoto University.

#### Acid treatment

Cells were treated with acid buffer according to previous reports.^39^^,^^43^ In brief, DCs were washed twice in PBS and resuspended for 4 min at 20°C in citrate buffer (0.133 M citric acid and 0.066 M Na_2_HPO_4_, pH3.3). The treatment was stopped by the addition of an excess of PBS containing 5% FCS. After being washed, DCs were stained with mAbs and analyzed by flow cytometry as described above.

#### *In vitro* antigen-presentation

UV (100 mJ/cm^2^)-irradiated splenocytes were loaded with OVA (1 mg/mL) by osmotic shock according to our previous report.^23^ UV (500 mJ/cm^2^)-irradiated OVA/B16 cells (3 × 10^4^ cells per well) were also used as dead cells. For masking PtdSer on apoptotic cells, these cells were pretreated with 1.2 μg/mL of soluble D89E protein for 30 min at 37°C. For preparation of naive CD8^+^ T cells, OT-I mouse splenocytes were stained with FITC-*anti*-CD8α mAb (clone 53–6.7, BioLegend), and CD8^+^ T cells were purified using anti-FITC-microbeads (130-048-701, Miltenyi Biotech). For the proliferation assay, CD8^+^ T cells were labeled with CellTrace CFSE Cell Proliferation kit for flow cytometry (C34554, Thermo Fisher Scientific) according to the manufacturer’s instructions. CFSE-labeled OT-I T cells (2 × 10^5^ cells per well), splenic DCs (1 × 10^5^ cells per well), and OVA-loaded apoptotic splenocytes (1 × 10^5^ cells per well) or apoptotic OVA/B16 cells (3 × 10^4^ cells per well) were co-cultured in 96-well flat-bottom cell culture plates. Two days later, cells were harvested and stained with PE-*anti*-TCR Vβ5.1/5.2 mAb (clone MR9-4, BioLegend) and analyzed on an Accuri C6 Plus cytometer (BD Bioscience). Production of IFN-γ in the culture supernatant was measured by ELISA (DY485, R&D systems) according to the manufacturer’s instructions. To address whether CD80 and CD86 are involved in IFN-γ production, DCs were pretreated with 1 μg/mL of mouse CTLA4-Ig (591806, BioLegend) or control human IgG (31154, Thermo Fisher Scientific) for 60 min.

#### *In vivo* antigen-presentation

CFSE-labeled OT-I T cells (2 × 10^6^ cells per mouse) were adoptively transferred into B6 WT or β2m KO mice. The following day, mice were injected intravenously with the indicated number of UV (100 mJ/cm^2^)-irradiated and OVA (5 mg/mL)-loaded WT or β2m KO mouse dead cells with PBS or low molecular weight (range 0.2 kb–1 kb) polyI:C (200 μg per mouse) (tlrl-picw, InvivoGen). Two days later, mice were sacrificed, and splenocytes were stained with PE-*anti*-TCR Vβ5.1/5.2 mAb (clone MR9-4, BioLegend). CFSE fluorescence intensity of TCR Vβ5.1/5.2^+^ cells was analyzed using FACSMelody (BD Biosciences) or Accuri C6 Plus cytometer (BD Biosciences). To address whether CD80 and CD86 are involved in *in vivo* OT-I T cell proliferation, mice were injected intraperitoneally with Abatacept (Bristol Myers Squibb) or human IgG (300 μg each per mouse).

In another model, mice receiving CFSE-labeled OT-I T cells as described above were injected subcutaneously with live, UV (500 mJ/cm^2^)-irradiated, or f-t SCT/BW5147 cells (2 × 10^6^ cells) into the footpad with i.p injection of PBS or polyI:C (200 μg per mouse). Three days later, mice were sacrificed, and the draining PLN cells and non-draining ALN cells were analyzed as described above.

### Quantification and statistical analysis

All quantitative data collected from experiments is represented as individual values (dots), mean (columns), and SD (error bars) as indicated in the figure legends. Statistical analyses were performed using one- or two-way ANOVA, or unpaired two-tailed Student’s t test: ∗*p* < 0.05; ∗∗*p* < 0.01.
