# Supplementary material for: MHC class I-dressing is mediated via phosphatidylserine recognition and is enhanced by polyI:C
Source: iScience. 2024 Apr 10;27(5):109704. doi: 10.1016/j.isci.2024.109704 (PMC11046299; doi:10.1016/j.isci.2024.109704)
Supplement: Document S1. Figures S1–S5 [file mmc1.pdf]

iScience, Volume *27*

Supplemental information

MHC class I-dressing is mediated via phosphatidylserine recognition and is enhanced by polyI:C

Arisa Hori, Saori Toyoura, Miyu Fujiwara, Ren Taniguchi, Yasutaka Kano, Tomoyoshi Yamano, Rikinari Hanayama, and Masafumi Nakayama

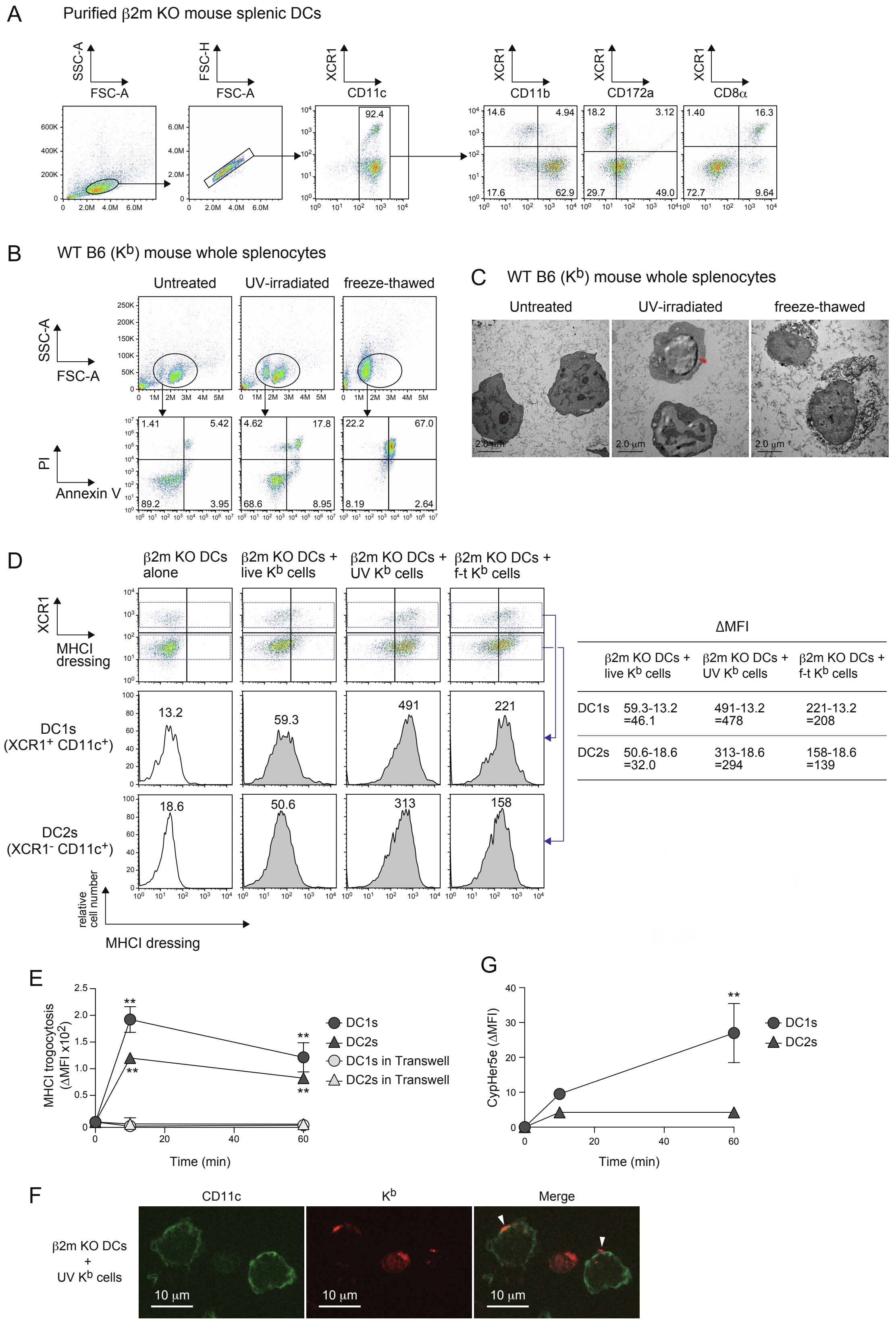

ΔMFI	β2m KO DCs + live Kb cells	β2m KO DCs + UV Kb cells	β2m KO DCs + f-t Kb cells
DC1s	59.3-13.2 =46.1	491-13.2 =478	221-13.2 =208
DC2s	50.6-18.6 =32.0	313-18.6 =294	158-18.6 =139

Figure S1. Cell preparation and MHCI dressing assay, Related to Figure 1.

(A) Purified β2m KO mouse splenic DCs were analyzed by flow cytometry with the indicated mAb staining.

(B, C) Untreated, UV-irradiated, and freeze-thawed (f-t) WT MHCI H-2K^b (K^b) mouse splenocytes were analyzed by flow cytometry with Annexin V/propidium iodide (PI) staining in (B) and by transmission electron microscopy (TEM). A red arrowhead indicates chromatin condensation in (C).

(D) DC1s and DC2s in Figure 1A were gated, and the median fluorescent intensity (MFI) of K^b was indicated in histograms. The ΔMFI was calculated by subtracting MFI of K^b on each subset from MFI of K^b on the untreated subset.

(E) β2m KO mouse splenic DCs (1x10^5 per well) were cultured with UV-irradiated K^b splenocytes (1x10^6 per well) for the indicated periods in 96-well flat-bottom cell culture plates or 96-transwell plates with a 0.4-μm pore membrane. K^b acquisition by DC1s (CD8α$^+$ CD11c$^+$ cells) and DC2s (CD8α$^-$ CD11c$^+$ cells) was analyzed by flow cytometry as in (D). Data are shown as mean ± SD (n=3 independent pools). **p < 0.01 compared with 0 min, by two-way ANOVA.

(F) β2m KO mouse splenic DCs were cultured with UV-irradiated WT K^b mouse splenocytes. Cells were stained with fluorescently labeled mAbs and were analyzed by fluorescence microscopy. White arrowheads indicate dead cell-derived K^b on DCs.

(G) β2m KO mouse splenic DCs were cultured with UV-irradiated and CypHer5E-labeled K^b splenocytes for the indicated periods in 96-well plates. The engulfment of dead cells (efferocytosis) by DC1s (CD8α$^+$ CD11c$^+$ cells) and DC2s (CD8α$^-$ CD11c$^+$ cells) was analyzed by flow cytometry as in (D). Data are shown as mean ± SD (n=3 independent pools). **p < 0.01 compared with 0 min, by two-way ANOVA.

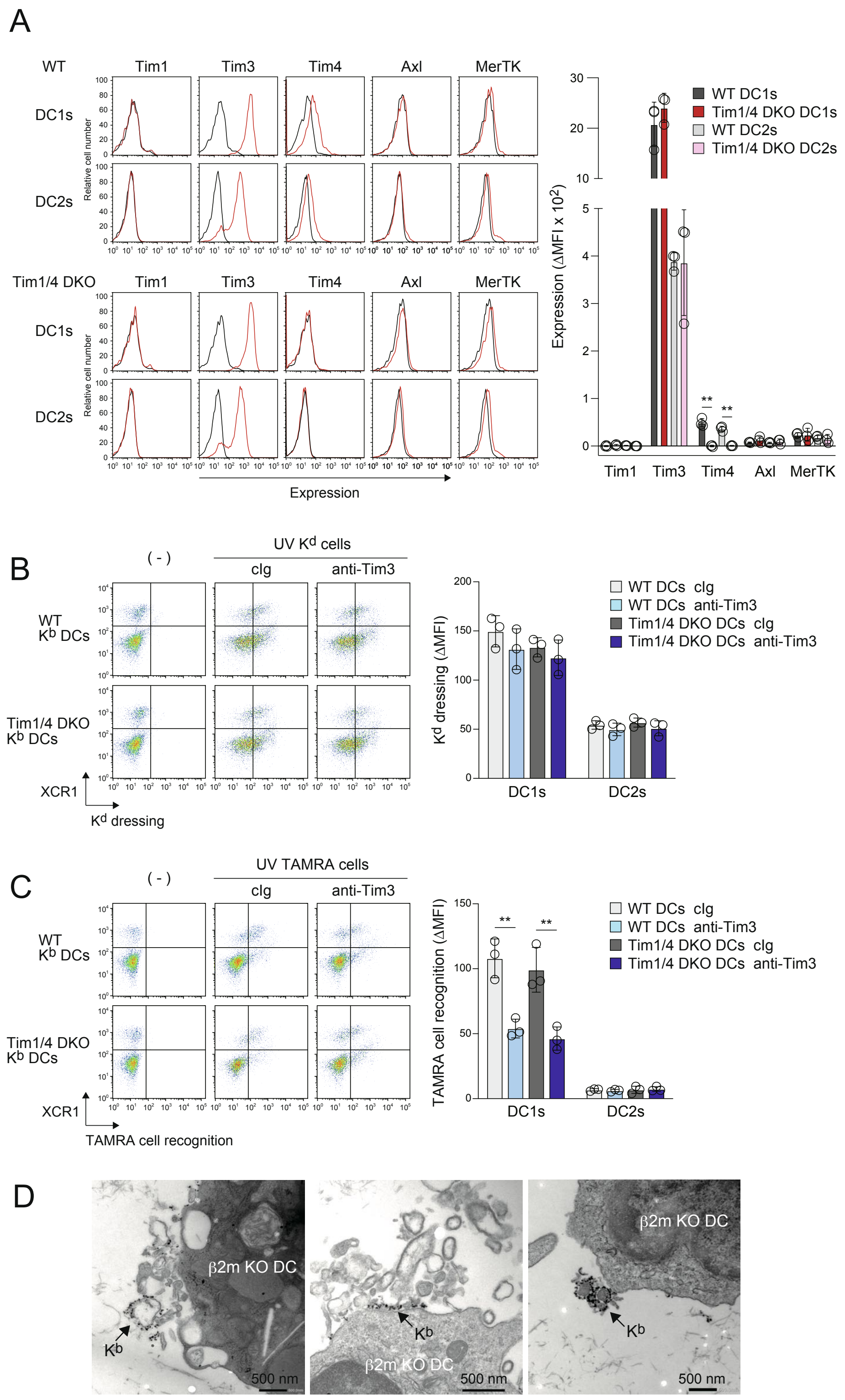

Figure S2. Mouse splenic DCs acquire MHCI from dead cells via Tim-3- and Tim4-independent pathway, Related to Figures 1 and 2.

(A) Tim1, Tim3, Tim4, Axl, and MerTK expression on C57BL/6 WT and Tim1/4 DKO mouse splenic DC1s (XCR1$^+$ CD11c$^+$ cells) and DC2s (XCR1$^-$ CD11c$^+$ cells) was analyzed by flow cytometry. Black histograms indicate the mixture of control rat IgG2a and IgG2b (cIg). Red histograms indicate anti-Tim1, -Tim3, -Tim4, -Axl, or -MerTK mAb. The graph indicates individual values (dots), mean (columns), and SD (error bars) from n=3 independent pools. **$p < 0.01$, two-way ANOVA.

(B) C57BL/6 WT and Tim1/4 DKO mouse splenic DCs (1x10^5) were cultured with UV-irradiated BALB/c (K^d) mouse splenocytes (5x10^5) in the presence of cIg or anti-Tim3 mAb in a microtube for 10 min. The K^d dressing on CD11c$^+$ cells was analyzed by flow cytometry as in Figure S1D. The graph indicates individual values (dots), mean (columns), and SD (error bars) from n=3 independent pools.

(C) C57BL/6 WT or Tim1/4 DKO mouse splenic DCs (1x10^5 each) were cultured with tetramethylrhodamine (TAMRA)-labeled and UV-irradiated splenocytes (5x10^5) in the presence of cIg or anti-Tim3 mAb for 60 min in a microtube. The recognition (binding and engulfment) of TAMRA-labeled cells by CD11c$^+$ cells was analyzed by flow cytometry as in Figure 1C, D. The graph indicates individual values (dots), mean (columns), and SD (error bars) from n=3 independent pools. **$p < 0.01$, two-way ANOVA.

(D) β2m KO mouse DCs cultured with UV-irradiated K^b splenocytes were stained with biotinylated anti-K^b, followed by streptavidin-10 nm gold particles. Cells were analyzed by TEM as in Figure 2F. Black arrows indicate K^b. Black bars indicate 500 nm.

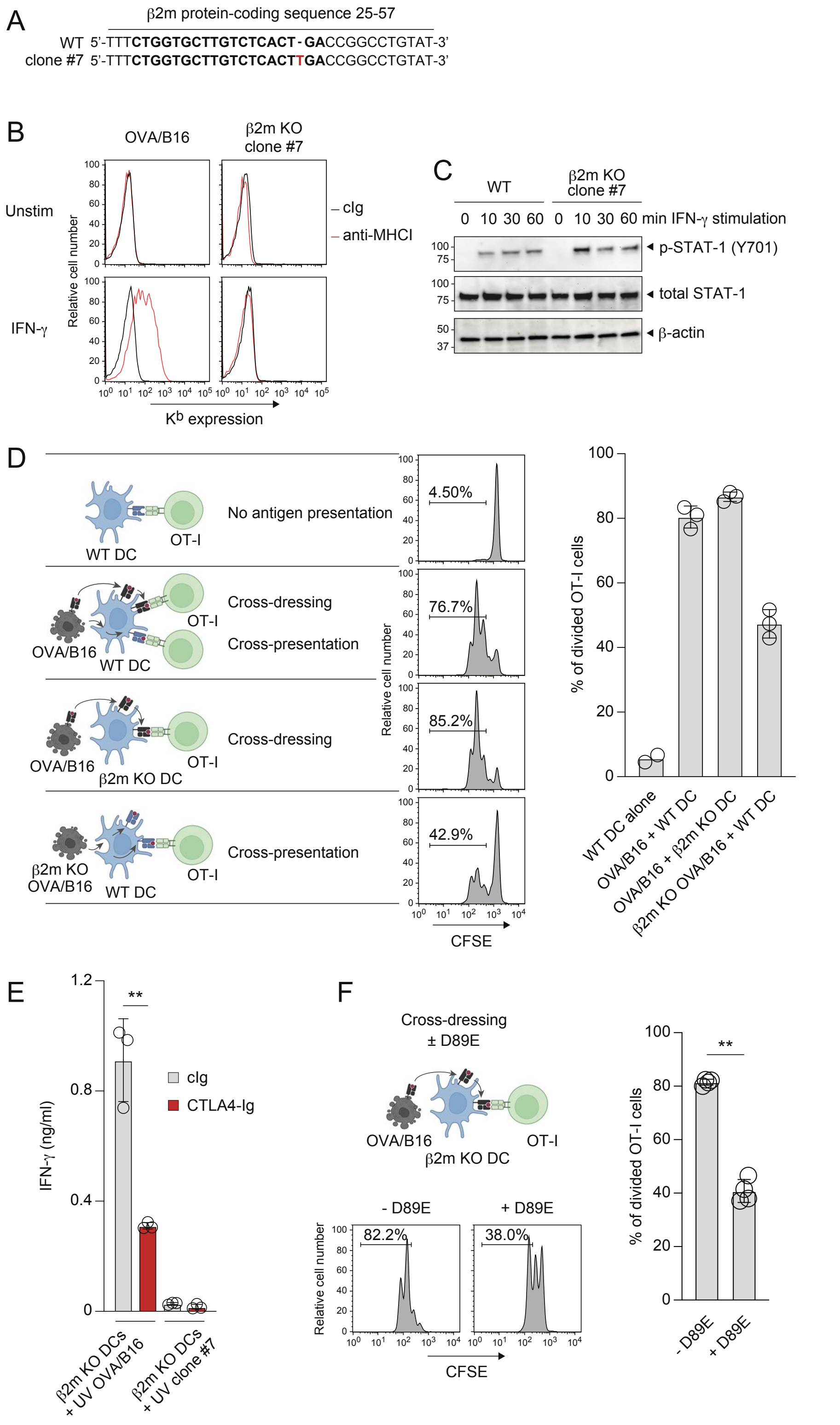

Figure S3. Cross-dressing of tumor cell-derived MHCI, Related to Figure 3.

(A) Sequence of β2m in WT B16 melanoma cell line and mutant clone #7 alleles around the target locus. The gRNA target sequence is shown in bold. An inserted base is in red.

(B) WT OVA/B16 cells and β2m KO OVA/B16 clone #7 cells were left unstimulated or were stimulated with IFN-γ. The K^b expression on these cells was analyzed by flow cytometry. Black and red lines indicate staining of cIg and anti-Kb mAb, respectively.

(C) Phosphorylation of STAT1 was analyzed by western blot.

(D) CFSE-labeled OT-I T cells were co-cultured with indicated splenic DCs with or without the indicated dead tumor cells for two days. OT-I T cell proliferation was analyzed by flow cytometry as in Figure 3B. Numbers in histograms indicate the percentage of divided OT-I T cells. The graph shows individual value (dots), mean (columns), and SD (error bars) (n=2 or 3 independent replicates).

(E) OT-I T cells were co-cultured with indicated DCs and apoptotic tumor cells in the presence of CTLA4-Ig or control human IgG (cIg). Production of IFN-γ was analyzed as in Figure 3C. Data are shown as individual value (dots), mean (columns), and SD (error bars) (n=3 independent replicates). **$p < 0.01$, one-way ANOVA.

(F) CFSE-labeled OT-I T cells were co-cultured with β2m KO splenic DCs and D89E-pretreated or untreated apoptotic tumor cells for two days. OT-I T cell proliferation was analyzed as in (D). Numbers in histograms indicate the percentage of divided OT-I T cells. The graph shows individual value (dots), mean (columns), and SD (error bars) (n=4 independent replicates). **$p < 0.01$, unpaired two-tailed Student's t test.

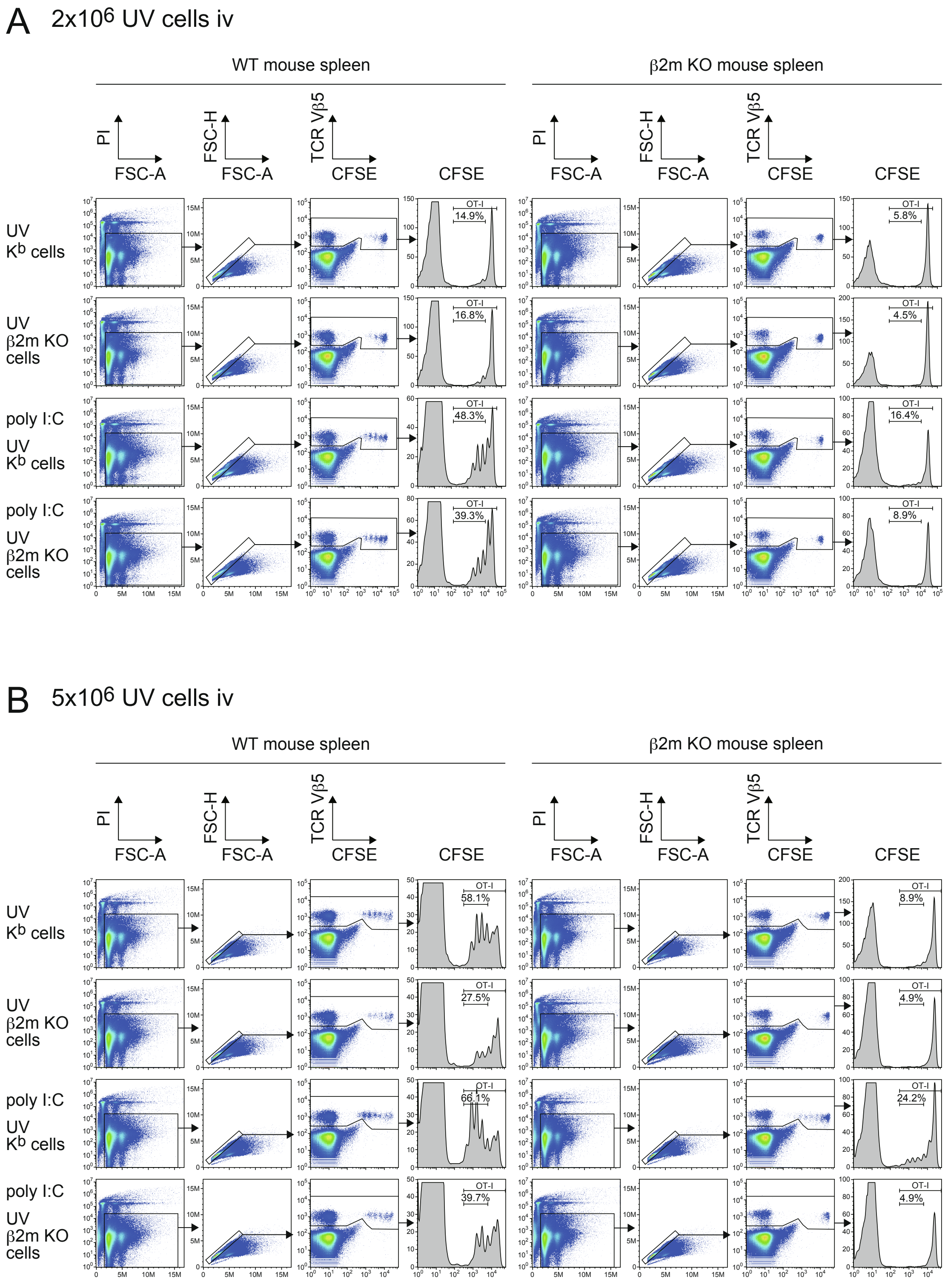

Figure S4. *In vivo* OT-I T cell proliferation assay and the gating strategy in Figure 4C.
(A, B) WT K^b or β2m KO mice adoptively transferred with CFSE-labeled OT-I T cells were injected i.v. with $2x10^6$ of OVA-loaded dead splenocytes in (A) and $5x10^6$ of these cells in (B), with PBS or polyI:C (200 μg/mouse). Two days later, OT-I T cell proliferation (CFSE intensity in TCR $V\beta5^+$ cells) in the spleen was analyzed by flow cytometry.

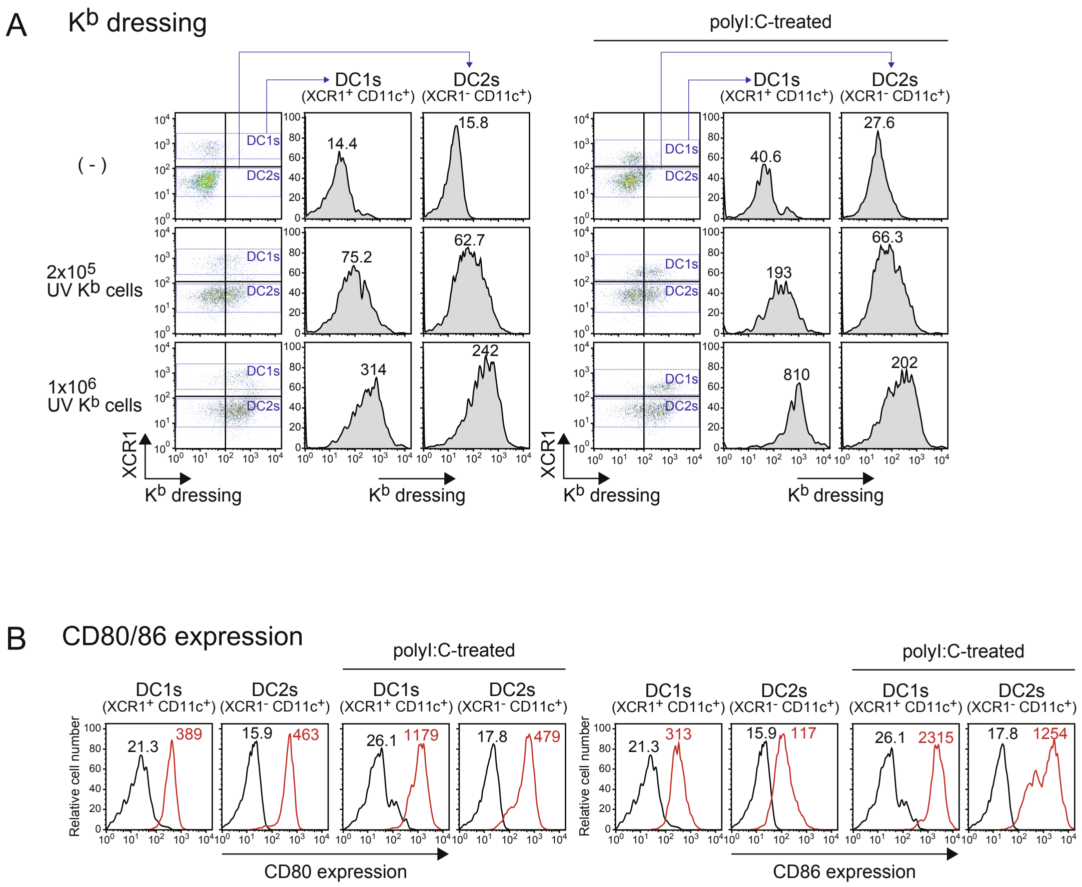

Figure S5. PolyI:C enhances MHCI dressing, Related to Figure 4D.
(A) β2m KO mice were injected intraperitoneally (i.p.) with PBS or polyI:C (200 μg/mouse). The following day, mouse splenic DCs were prepared and ($1x10^5$ each) were cultured with the indicated number of UV-irradiated K^b splenocytes for 10 min in a microtube. DC ($CD11c^+$ cell) K^b acquisition was analyzed by flow cytometry. (B) Splenic DCs were prepared from mice treated as in (A). The expression of CD80 and CD86 on the indicated DCs was analyzed by flow cytometry using anti-CD80 or -CD86 mAb (red histograms), and cIg (black histograms) in (D). Numbers in histograms indicate the MFI.
